# The clinical utility and cost-effectiveness of routine vitamin B_12_ screening in adult psychiatric patients

**DOI:** 10.4102/sajpsychiatry.v31i0.2449

**Published:** 2025-09-08

**Authors:** Tracy A. Hollander, Vidette M. Juby

**Affiliations:** 1 Department of Psychiatry, College of Clinical Medicine, University of KwaZulu-Natal, Durban, South Africa

**Keywords:** Vitamin B_12_ deficiency, cobalamin deficiency, vitamin B_12_ screening, adult psychiatric patients, clinical utility, cost-effectiveness

## Abstract

**Background:**

Diagnosing and treating vitamin B_12_ deficiency in psychiatric populations is important, but the justification for routine screening in patients without risk factors or physical findings remains uncertain, especially in resource-limited settings.

**Aim:**

This study aimed to assess the clinical utility and cost-effectiveness of routine vitamin B_12_ screening in adult psychiatric inpatients.

**Setting:**

The study was conducted at Townhill Hospital, a tertiary psychiatric facility in South Africa.

**Methods:**

A retrospective chart review was performed for the period 01 July 2021 to 31 December 2022. Data collected included demographics, clinical diagnoses, medications, risk factors for deficiency, vitamin B_12_ test results, associated costs, and clinical responses to abnormal findings.

**Results:**

Of 366 patients (168 male, 198 female; mean age 35.95 ± 13.44 years), the mean serum vitamin B_12_ level was 423.86 mmol/L (SD ± 233.37), with a median of 359 mmol/L. Vitamin B_12_ deficiency was identified in eight patients (2.2%). The cost per deficient patient was R5780.73. Statistically significant associations were found between low B_12_ levels and pregnancy, vegetarian diet, abdominal surgery, and metformin use (*p* < 0.05). Only half of the deficient patients received replacement therapy.

**Conclusion:**

Routine vitamin B_12_ screening in the absence of physical findings or known risk factors is not clinically or economically justified. Targeted screening should be considered to optimise resource use and patient outcomes.

**Contribution:**

In resource-constrained environments, prioritising high-yield interventions is essential to improving care efficiency.

## Introduction

Cobalamin (vitamin B_12_) is a water-soluble vitamin obtained by humans from animal sources, including meat, dairy products and eggs.^1^ This vitamin is vital in deoxyribonucleic acid (DNA) synthesis, red blood cell formation and neurological functioning.^2^ It is also linked to the synthesis of various neurotransmitters, and thus, it has been implicated in the pathogenesis of various psychiatric disorders.^3^

Deficiency presents with broad, non-specific clinical features, including symptoms related to anaemia, gastrointestinal issues, neurological impairments and psychological disturbances.^4^ Often, neuropsychiatric symptoms are the first clinical manifestation and precede the haematologic and gastrointestinal symptoms.^5,6,7^ Psychiatric manifestations of vitamin B_12_ deficiency can include depression, apathy, irritability, dementia, mania, catatonia, delirium, anxiety and psychosis.^8,9,10^

The prevalence of low serum or plasma vitamin B_12_ concentrations among adults varies, with rates of 2.7% in the United States, 8.3% in the United Kingdom and 14.7% in Germany.^11^ In Africa, available data on prevalence indicate that 35% and 15% were deficient in Kenya and Ethiopia, respectively; however, in Ghana, the prevalence was relatively lower (7%).^12^ Epidemiological data for cobalamin deficiency in South Africa are limited.

Psychiatric patients are frequently screened for vitamin B_12_ deficiency in the absence of haematologic or neurologic findings,^13^ with some studies going as far as recommending that routine screening for serum vitamin B_12_ levels should be adopted by all hospitals for psychiatric patients.^14,15^ Based on the authors’ clinical experience, routine screening for vitamin B_12_ deficiency is common practice across psychiatric departments in KwaZulu-Natal, including at Townhill Hospital. At this facility, admitted patients are typically screened for vitamin B_12_ deficiency, irrespective of the presence of specific risk factors.

Certain groups are at heightened risk for vitamin B_12_ deficiency because of factors that impair intake or absorption. These include individuals with restricted diets, pregnant women, the elderly, people with gastritis and certain autoimmune disorders (e.g., pernicious anaemia), people with intestinal diseases (e.g., Crohn’s, celiac disease), those with surgeries involving the gastrointestinal tract and people on medications that inhibit B_12_ absorption, such as metformin, proton pump inhibitors (PPIs), H_2_-receptor antagonists and oral contraceptives.^4,16,17,18,19^

A diagnosis of vitamin B _12_ deficiency is challenging, as confirmation relies on laboratory findings, which remain controversial. In terms of laboratory investigations for B _12_ deficiency, there are four biological markers of B _12 _: total serum vitamin B _12 _, holotranscobalamin (holoTC) and measures of B _12_ metabolites homocysteine (Hcy) and methylmalonic acid (MMA). Each of these tests have limitations. ^4^ The serum B _12_ is the most frequently used test and it measures the circulating concentration of vitamin B _12_ bound to both vitamin binding proteins, transcobalamin and haptocorrin. ^20^ A critical problem with these assays is that they can be influenced by the presence of interfering anti-intrinsic antibodies (particularly in patients with pernicious anaemia), thereby giving elevated vitamin B _12_ concentrations in cobalamin-deficient patients. ^21^ There is also a need for scientific consensus defining cut-off values to diagnose deficiency states. ^22^ Measuring Hcy and MMA is based on the rationale that cobalamin deficiency leads to the accumulation of these metabolites. ^23^ While these tests may have greater sensitivity for cobalamin deficiency, they remain problematic in diagnosing B _12_ deficiency. Differential causes need to be considered if MMA levels are elevated. Other possible causes of elevated MMA include renal impairment or dehydration, ^24^ and if Hcy is elevated, it may reflect folate deficiency; therefore, this would have to be excluded. ^25^ Therefore, vitamin B _12_ deficiency can be suspected only when serum levels are low, and MMA and Hcy are elevated without renal disease, volume depletion and folate deficiency. A variety of conditions may impact serum cobalamin levels, but MMA or Hcy level within the normal range suggests that vitamin B _12_ deficiency can be excluded. ^26^ Measuring holoTC, the bioactive form of vitamin B _12 _, remains debatable, with one large study concluding that holoTC has the highest diagnostic accuracy in predicting deficiency ^27^ and another extensive study showing that the holoTC immunoassay cannot be used to reliably determine the vitamin B _12_ status. ^28^ In our setting, the serum vitamin B _12_ is the most cost-effective test and widely used test to assess for cobalamin deficiency.

Clinical utility is increasingly utilised in health care, and critical measures of clinical utility include clinical effectiveness and cost-effectiveness. However, as Smart highlights, this narrow focus overlooks other essential factors that influence clinical decision-making, including appropriateness, accessibility, practicality and acceptability, all of which should be considered when assessing clinical utility.^29^ Cost-effectiveness analysis (CEA) assesses the outcomes and costs of various strategies or interventions.^30^

While diagnosing and treating vitamin B_12_ deficiency in the psychiatric population is essential, the prevalence of this condition among patients admitted to Townhill Hospital remains unclear. Consequently, it is uncertain whether routine screening is justified in non-vulnerable patients who lack haematological or neurological findings. Furthermore, current testing practices may need to be more reliable, potentially contributing to unnecessary costs in an already resource-constrained environment. This study investigated the clinical utility and cost-effectiveness of routine screening for vitamin B_12_ deficiency in adult psychiatric patients admitted to Townhill Hospital.

## Research methods and design

### Study design

This study was a quantitative descriptive analysis and data were collected retrospectively from existing medical records.

### Study setting

The study was conducted at Townhill Hospital, a tertiary-level facility in Pietermaritzburg, South Africa. Townhill Hospital is a specialist psychiatric institution providing general and tertiary psychiatric services and inpatient and outpatient care. Serving a broad catchment area, the hospital accepts mental health referrals from any health care establishment within the KwaZulu-Natal province.

### Study population and sampling

All adult inpatients (≥ 18 years) admitted to Townhill Hospital between 01 July 2021 and 31 December 2022 were eligible for inclusion if their serum vitamin B_12_ levels were measured either within 3 months prior to admission or during their hospital stay.

Exclusion criteria included patients younger than 18 years, those whose vitamin B_12_ levels were assessed more than 3 months before admission and cases with missing clinical records or unavailable B_12_ results.

### Data collection

Patient records were reviewed to assess sociodemographic factors that could influence vitamin B_12_ levels. In addition, clinical data such as psychiatric history and discharge diagnoses were documented. The total number of vitamin B_12_ tests performed, their yield and the record of action taken following an abnormal result were recorded. The cost of serum vitamin B_12_ testing was also recorded. The cut-off that the National Health Laboratory Service (NHLS) uses to define low vitamin B_12_ levels is less than 156 mmol/L. This value was employed to identify vitamin B_12_ deficiency in the study participants.

### Data analysis

Data analyses were performed using SPSS version 29. Statistics for continuous data (numerical data) included measures of central tendency and data were interpreted as a mean ± standard deviation (SD) unless stated otherwise. One-way ANOVA and t-tests were used to compare continuous variables between different groups. Relationships between categorical variables were tested using the Chi-square test. Statistical significance was accepted at *p* ≤ 0.05.

### Ethical considerations

Data were de-identified to protect participant identities. The data-collection procedures involved only a retrospective file review, and no patient intervention or interaction occurred. Ethical clearance to conduct this study was obtained from the University of KwaZulu-Natal Biomedical Research Ethics Committee (No.BREC/00006699/2024). The study was conducted in accordance with the Medical Research Ethical Guidelines on Human Research of the Department of Health 2004.

## Results

A total of 496 adult patients (18 years and older) were admitted to Townhill Hospital during the study period. Out of the total sample, 81 participants (16.3%) were excluded from the study because of the absence of documented serum vitamin B_12_ levels in their medical records. In addition, 49 participants (9.9%) were excluded because their files were unavailable at the hospital registry department. The final sample thus consisted of 366 (73.8%) cases.

Key sociodemographic and clinical characteristics are summarised in Table 1.

**TABLE 1:** Sociodemographic and clinical characteristics (***N*** = 366).

Variable	Category	*n*	%
Gender	Female	198	54.1
	Male	168	49.9
Age (in years)	18–24	87	23.8
	25–39	161	44.0
	40–59	86	23.5
	60+	32	8.7
Race	African people	275	75.1
	Indian people	48	13.1
	Caucasian people	37	10.1
	Mixed race people	6	1.6
Pregnancy status	Pregnant	4	1.1
	Not pregnant	184	50.3
	Not documented	10	2.7
	Not applicable (male)	168	45.9
Dietary preference	Regular	290	79.2
	Vegetarian	12	3.3
	Not documented	64	17.5
Psychiatric history	Index presentation	79	21.6
	Previous history	287	78.4
Medical illness	HIV	76	20.8
	Diabetes	27	7.4
	Pernicious anaemia	0	0
	Gastritis	0	0
	Crohn’s disease	0	0
	Celiac disease	0	0
	Hypothyroidism	15	4.1
Surgical history	Abdominal surgery	17	4.6
	Non-abdominal surgery	81	22.1
	No surgical history	206	56.3
	Not documented	60	16.4
Alcohol use	Alcohol use reported	193	52.7
	No reported use	147	40.7
	Not documented	22	6
	Alcohol use disorder diagnosed	47	12.8
Clinical diagnosis	Schizophrenia	118	32.2
	Schizoaffective disorder	67	18.3
	Major depressive disorder	34	9.3
	Bipolar 1 disorder	65	17.8
	Bipolar 2 disorder	2	0.5
	Psychotic disorder because of another medical condition	28	7.7
	Substance-induced psychotic disorder	6	1.6
	Mood disorder because of another medical condition	6	1.6
Medication use	Antipsychotic agents	342	93.4
	Mood stabilisers	191	52.1
	Antidepressant agents	86	23.5
	Antiretroviral agents	76	20.8
	Metformin	26	7.1
	Proton pump inhibitors	11	3.0
	H_2_ receptor antagonists	0	0
	Oral contraceptives	0	0

HIV, human immunodeficiency virus.

The mean age was 35.95 years (SD ± 13.44). Most patients were female (*n* = 198, 54.1%), and 45.9% (*n* = 168) were male. The study population predominantly comprised African patients, accounting for 75.1% of the cohort (*n* = 275).

Four of the patients (1.1%) were pregnant.

Most of the participants (*n* = 290, 79.2%) reported following a regular omnivorous diet, while 12 patients (3.3%) were identified as vegetarian. The dietary preference information was missing for 64 patients (17.5%).

The majority of the participants, totalling 78.4% (*n* = 287), were established mental health care users, while for 79 individuals (21.6%), it was their first contact with mental health services.

In this study population, 76 patients (20.8%) had a diagnosis of human immunodeficiency virus (HIV), with other common comorbidities including hypertension (*n* = 60, 16.4%), dyslipidaemia (*n* = 35, 9.6%), diabetes (7.4%) and epilepsy (*n* = 26, 7.1%). A total of 193 patients (52.7%) reported alcohol consumption, of whom 12.8% (*n* = 12.8%) were diagnosed with alcohol use disorder. The primary psychiatric diagnoses included schizophrenia (*n* = 118, 32.2%), schizoaffective disorder (*n* = 67, 18.3%) and bipolar 1 disorder (*n* = 65, 17.8%), with a notable prevalence of cannabis use disorder (*n* = 47, 17.8%) and multiple substance use disorders (*n* = 47, 12.8%). Antipsychotics were the most prescribed medication (*n* = 342, 93.8%), followed by mood stabilisers (*n* = 191, 52.2%), antidepressants (*n* = 86, 23.5%) and antiretrovirals (*n* = 76, 20.8%). Other medications included metformin (*n* = 26, 7.1%) and PPIs (*n* = 11, 3.0%), with no use of oral contraceptives or H2-receptor antagonists in this population. A total of 81 patients (22.1%) had a history of prior non-abdominal surgery, while 17 (4.6%) had a history of prior abdominal surgery.

Key variables related to the testing of vitamin B_12_ are outlined in Table 2.

**TABLE 2:** Vitamin B_12_ testing (***N*** = 366).

Variable	Value
Mean serum vitamin B_12_ level	423.86 mmol/L (± 233.37)
Number of participants with vitamin B_12_ deficiency	8 (2.2%)
Total cost of the serum vitamin B_12_ tests	R46 245.80
Cost-utility (total cost per patient with vitamin B_12_ deficiency)	R5780.73

The mean serum vitamin B_12_ level was 423.86 mmol/L (SD ± 233.37). The interquartile range (IQR) was 243 mmol/L, with values ranging from 118 mmol/L to 1476 mmol/L. The median serum vitamin B_12_ concentration was 359 mmol/L. In this study, eight (2.2%) patients had low vitamin B_12_ levels. Only one (0.3%) of these patients had a Hcy level test performed, and none received an MMA test. Most of the study sample (*n* = 358, 97.8%) had normal serum vitamin B_12_ levels.

There was a statistically significant association between low vitamin B_12_ levels and both pregnancy and dietary status, with more of these patients having a low vitamin B_12_ level (*p* < 0.05). However, although these were statistically significant, the effect sizes were small (phi = 0.18 and 0.15, respectively), and the assumption of expected cell counts exceeding five was violated in the pregnancy group.

There was no statistically significant association between mean age and vitamin B_12_ deficiency, including no statistically significant difference in the percentage of those with low vitamin B_12_ levels and advanced age of > 60 years (*p* > 0.05).

Associations of low vitamin B_12_ with sociodemographic variables are outlined in Table 3.

**TABLE 3:** Associations of low vitamin B_12_ with sociodemographic variables.

Variable	Mean vitamin B_12_ level	ANOVA results		Vitamin B_12_ level		Chi-square results	
		*F*-statistic	*p*	Low (< 156 mmol/L) %	Normal %	*χ*^2^-value	*p*
**Gender**							
Male	417.0	0.37	0.54	3	97	1.44	0.23
Female	432.9	-	-	1	99	-	-
**Race**							
African people	426.2	0.22	0.88	2	98	1.84	0.61
Indian people	413.19	-	-	0.4	99/6	-	-
Caucasian people	431.08	-	-	0	0	-	-
Mixed race people	356.83	-	-	0	0	-	-
**Pregnant**							
Yes	416.0	0.19	0.90	25	75	6.29	**0.01**
No	415.54	-	-	3	97	-	**-**
**Dietary preference**							
Vegetarian	332.83	1.17	0.32	8	92	6.54	**0.01**
Regular	430.46	-	-	1	99	-	**-**
**Previous psychiatric history**							
Yes	419.62	0.44	0.51	2	98	0.40	0.53
No	439.25	-	-	1	99	-	-

Note: Statistical significance is indicated in bold text.

A statistically significant lower mean serum vitamin B_12_ level was observed in individuals with schizophrenia (*t* = 2.57, *p* = 0.01). However, no significant association was found between a diagnosis of schizophrenia and vitamin B_12_ deficiency (χ^2^ = 0.14, *p* > 0.05).

A general trend of a lower mean serum vitamin B_12_ level in patients with comorbidities was seen, but this was not statistically significant (*p*-values > 0.05). However, there was a statistically significant difference regarding patients with diabetes, with more of these patients having a low vitamin B_12_ level (χ^2^ = 3.72, *p* = 0.05).

There was no statistical difference between patients on chronic medication and mean serum vitamin B_12_ levels (*p*-values > 0.05). However, there was a statistically significant difference with regard to patients using metformin, with more of these patients having a deficient vitamin B_12_ level (χ^2^ = 3.97, *p* = 0.05).

The results showed no statistical differences between patients with surgical history, alcohol use or alcohol use disorder and mean serum vitamin B_12_ levels (*p*-values > 0.05). However, there was a statistically significant difference regarding patients with a history of abdominal surgery, with more of these patients having a low vitamin B_12_ level (χ^2^ = 6.19, *p* = 0.05).

Associations of low vitamin B_12_ with important clinical variables are summarised in Table 4a and Table 4b.

**TABLE 4a:** Associations of low vitamin B_12_ and important clinical variables.

Variable	Mean vitamin B_12_ level	*t*-test results		Vitamin B_12_ level		Chi-square results	
		*t*-value	*p*	Low (< 156 mmol/L)	Normal	*χ*^2^-value	*p*
**HIV**							
No	417.25	−0.90	0.37	2	98	0.09	0.77
Yes	449.05	-	-	3	97	-	-
**Diabetes**							
No	427.30	1.00	0.32	2	98	3.72	**0.05**
Yes	380.63			7	93	-	**-**
**Metformin**							
No	427.75	1.15	0.25	2	98	3.97	**0.05**
Yes	372.96	-	-	8	92	-	**-**
**PPI**							
No	421.20	−1.24	0.22	2	98	0.25	0.62
Yes	509.45	-	-	0	100	-	-

Note: Statistical significance is indicated in bold text.

HIV, human immunodeficiency virus; PPI, proton pump inhibitors.

**TABLE 4b:** Associations of low vitamin B_12_ and important clinical variables.

Variable	Mean vitamin B_12_ level	ANOVA results		Vitamin B_12_ level		Chi-square results	
		*F*-statistic	*p*	Low (< 156 mmol/L)	Normal	*χ*^2^-value	*p*
**Alcohol use disorder**							
No	419.96	0.29	0.59	3	97	1.21	0.27
Yes	439.38	-	-	0	100	-	-
**Surgical history**							
No surgical history	412.4	0.99	0.37	2	98	6.19	**0.05**
Previous abdominal surgery	354.94	-	-	12	88	-	**-**
Previous non-abdominal surgery	436.25	-	-	1	99	-	**-**

Note: Statistical significance is indicated in bold text.

Overall, three of the patients with low vitamin B_12_ had no identified risk factors (38%), while the remainder had at least one risk factor (*n* = 5, 62%). Two of the deficient patients had two or more risk factors.

The frequency of risk factors in patients with vitamin B_12_ deficiency is summarised in Table 5.

**TABLE 5:** The frequency of risk factors in patients with vitamin B_12_ deficiency (***N*** = 8).

Risk factor	Frequency	%
No risk factors	3	38
Age 60 years and older	1	12
On metformin	1	12
Pregnant	1	12
Vegetarian and abdominal surgery	1	12
Age 60 years and older, on metformin and abdominal surgery	1	12

### Treating vitamin B_12_ deficiency

Only four out of the eight patients with vitamin B_12_ deficiency received vitamin B_12_ replacement. One patient received an oral replacement and the other three received intramuscular supplementation.

### Cost of serum vitamin B_12_ testing

The total cost of serum vitamin B_12_ testing performed on the 366 included participants during the 18-month study period was R46 245.80.

### Cost-utility

The cost-effectiveness ratio (CER) was determined using Equation 1 (outcome = a positive vitamin B_12_ deficiency result):


$$\begin{array}{l} \text{CER}=\frac{\text{Totalcost}}{\text{Totaloutcome}}\\ =\frac{46245.80}{8} \end{array}$$ [Eqn 1]

the total cost per patient with low vitamin B_12_ levels was R5780.73.

## Discussion

This study investigated the clinical utility and cost-effectiveness of screening for vitamin B_12_ deficiency in adult psychiatric patients admitted to Townhill Hospital. This is the first documented analysis of the clinical utility and cost-effectiveness of routine vitamin B_12_ screening in a South African inpatient psychiatric population. Routine laboratory screening of psychiatric patients is widely practiced, primarily to detect occult medical conditions. It also facilitates the identification of underlying medical illnesses that may exacerbate or mimic psychiatric symptoms. Adequate screening tests should meet criteria for acceptability, suitability for the population and cost-effectiveness and should positively impact patient outcomes.^31^

### The number of patients screened for vitamin B_12_ deficiency

During the study period, 496 adult patients were admitted to Townhill Hospital. Among these, routine serum vitamin B_12_ screening tests were requested in at least 73.8% (*n* = 366) of this population.

### The prevalence of patients with vitamin B_12_ deficiency

In this study, eight participants (2.2%) had vitamin B_12_ deficiency. This rate contrasts with international prevalence data as the reported prevalence of vitamin B_12_ deficiency in patients admitted for psychiatric illness is between 5% and 30%.^10,15,32,33^ Available general population data from Africa estimate rates of 35% in Kenya, 15% in Ethiopia and 7% in Ghana.^12^

There remains no universally accepted cut-off for the diagnosis of vitamin B_12_ deficiency.^22,34^ In a review examining cut-off values, 13 authors used a threshold of 150 mmol/L to define deficiency, while 12 others reported 148 mmol/L as the standard.^34^ Consequently, the NHLS cut-off of < 156 mmol/L aligns closely with internationally referenced standards, making it unlikely that this threshold contributed to the unexpectedly low rate of deficiency observed.

Several factors may have contributed to this study’s lower prevalence of deficiency. One notable factor is the limited representation of individuals with established risk factors for deficiency. Risk factors for cobalamin deficiency include pregnancy, conditions that impair B_12_ absorption, certain medications, a history of abdominal surgery and advanced age.^4^ In this study, only a small percentage of patients fell into these categories: 1.1% (*n* = 4) were pregnant, none had conditions strongly linked to reduced B_12_ absorption (such as pernicious anaemia, gastritis, Crohn’s disease and celiac disease) and a minority were on medications that can affect B_12_ levels (7.1% on metformin, 3.0% on PPIs and none were on H_2_-receptor antagonists or oral contraceptives). In addition, only 4.6% (*n* = 17) reported a history of abdominal surgery, and 8.7% (*n* = 32) were older than 60.

Inadequate dietary intake is another significant risk factor for vitamin B_12_ deficiency.^4^ The dietary intake of B_12_ in both the general South African population and our psychiatric population may be higher than other African populations. A review of dietary surveys of in the South African population from 2000 to 2015 showed that chicken meat, eggs and full cream milk are among the 10 most frequently consumed foods in this population.^35^ Epidemiological data for cobalamin deficiency in South Africa are scarce; however, inadequate vitamin B_12_ intake in South Africa was demonstrated to be only 4.5% in 2017, and B_12_ supplies in the diet in Northern, Central and Southern Africa are considerably higher than in Western and Eastern Africa regions.^12^ Financial assistance may lower deficiency risk by supporting access to a more vitamin B_12_-rich diet, as demonstrated in a study in Kenya, where the Hunger Safety Net Programme, a cash transfer initiative, led to a 36.6% improvement in vitamin B_12_ intake after 24 months.^36^ Given that the majority of study participants were known mental health care users (*n* = 287, 78.4%) and may have been receiving financial support through a disability grant, their access to animal products in the diet could be higher than that of a substantial portion of the population in South Africa and other parts of the continent who lack any form of financial income.

Given the low prevalence of vitamin B_12_ deficiency observed among the study participants, the acceptability and appropriateness of routine screening for this deficiency in psychiatric patients without identifiable risk factors or physical findings warrant reconsideration.

### Associations of low vitamin B_12_ levels with sociodemographic and clinical variables

A statistically significant difference was observed in the prevalence of low vitamin B_12_ levels based on pregnancy status and dietary habits, with higher rates of deficiency in pregnant patients and those following a vegetarian diet (*p* < 0.05). This finding is unsurprising, as these factors are well-established risk factors for vitamin B_12_ deficiency.^4,16,17^ However, it is essential to note that, despite statistical significance, the effect sizes were small. Furthermore, there was a statistically significant difference between patients with diabetes (χ^2^ = 3.72, *p* = 0.05) and on metformin (χ^2^ = 3.97, *p* = 0.05), with more of these patients having a low vitamin B_12_ level. The association between diabetes and low vitamin B_12_ is likely attributable to the high proportion of diabetic patients prescribed metformin, a medication known to cause vitamin B_12_ deficiency by impairing B_12_ absorption.^19^ There was also a statistically significant difference regarding patients with abdominal surgery (χ^2^ = 6.19, *p* = 0.05), with more of these patients having vitamin B_12_ deficiency. Abdominal surgery is a well-known risk factor for cobalamin deficiency, as procedures involving any part of the gastrointestinal tract can result in decreased absorption of vitamin B_12_.^4^

These findings highlight the importance of screening for cobalamin deficiency in individuals with established risk factors.

### Treating patients with vitamin B_12_ deficiency

A study by Obeid et al. reviewed literature published over the past two decades to establish a consensus on managing vitamin B_12_ deficiency. They concluded that, ‘regardless of the cause of the deficiency, initial treatment with parenteral B_12_ was regarded as the first choice for patients with acute and severe manifestations of B_12_ deficiency’.^37^ The Standard Treatment Guidelines and Essential Medicines List for South Africa also recommends treating deficiency with parental cobalamin.^38^ In this study, of the patients with confirmed vitamin B_12_ deficiency (*n* = 8), three received appropriate parenteral supplementation, while one received only oral supplementation. Therefore, only half (*n* = 4) of those screened and identified as deficient benefited from the screening process.

### Screening test for Vitamin B_12_ deficiency

The National Institute for Health and Care Excellence (NICE) recommends performing a diagnostic test (a serum MMA) if screening tests such as serum B_12_ level or plasma Hcy are low before deciding if deficiency is likely.^39^ In our study, one (0.3%) of these patients had an Hcy level performed, and none received an MMA test. While markers such as MMA and Hcy may be valuable aids in diagnosing vitamin B_12_ deficiency, these tests are costly. Furthermore, these tests have their limitations.^24,25^ These limitations, combined with the costs associated with further testing in a resource-constrained setting, raise questions about the clinical utility of using MMA and Hcy to confirm a vitamin B_12_ deficiency diagnosis. The authors believe that, in this context, it was appropriate to treat vitamin B_12_ deficiency based on the initial screening test alone.

### Clinical utility

Important measures of clinical utility include the clinical effectiveness and cost-effectiveness of the particular test or intervention.^29^

In our cost-utility analysis concerning patients with low vitamin B_12_ levels, the total cost per patient was calculated to be R5780.73. This figure is notably high, particularly in a resource-constrained environment with limited financial resources. Furthermore, less than half of the patients diagnosed with vitamin B_12_ deficiency received appropriate treatment. This inadequate treatment rate contributes to an increased cost–utility ratio, as untreated deficiencies may lead to additional health care expenses and adverse health outcomes.

A clinically effective intervention can be characterised by several key attributes, including (but not limited to) the restoration of wellness, a sustained reduction in disease symptoms and a positive impact on the patient.^40^ In this study, given that less than half of the patients who screened positive for vitamin B_12_ deficiency received adequate treatment, most patients did not fulfil these essential attributes.

### Limitations

As this study was a retrospective chart audit, it was influenced by the quality of record-keeping, which sometimes limited the availability of comprehensive sociodemographic and clinical patient data. Furthermore, it was impossible to ascertain the recorded data’s accuracy.

In addition, the study was conducted at a single hospital, so the findings may not be generalisable to all psychiatric patients or other geographic regions.

The low prevalence of vitamin B_12_ deficiency within the sample represented a significant limitation, as it precluded the use of logistic regression to adjust for potential confounding variables in the statistical analysis.

Townhill Hospital, a tertiary facility, faces long admission waiting lists, causing delays of weeks or months for patient transfers. In cases where serum vitamin B_12_ levels were measured at Townhill instead of the referral hospital, receiving a balanced diet as an inpatient during the wait could affect the serum vitamin B_12_ results.

Moreover, it is impossible to determine the exact costs associated with consumables (such as syringes, needles, gloves and alcohol swabs); therefore, these costs were not included in the overall testing expenses. Consequently, the total cost of testing may be underreported.

## Conclusion

In our study, only 2.2% of individuals who underwent routine screening for vitamin B_12_ deficiency were found to have low cobalamin levels. The total cost per patient with vitamin B_12_ deficiency was R5780.73, a notably high figure. Additionally, less than half of the patients diagnosed with vitamin B_12_ deficiency received appropriate treatment, contributing to an increased cost-utility ratio. Consequently, our findings do not support routine screening for vitamin B_12_ deficiency, as it is not cost-effective.

The study identified a statistically significant difference in the prevalence of low vitamin B_12_ levels with established risk factors for cobalamin deficiency. Screening for vitamin B_12_ deficiency should be guided by clinical history (including inquiries about well-known risk factors) and physical findings. We recommend limiting ‘routine testing’ for deficiency to individuals exhibiting physical signs suggestive of deficiency, mental health care users not responding to treatment, and specific high-risk groups. These groups include pregnant women, the elderly, individuals on medications associated with vitamin B_12_ deficiency (such as metformin, PPIs, H_2_-receptor antagonists and oral contraceptives), those with conditions strongly linked to deficiency (such as Crohn’s disease, celiac disease and pernicious anaemia), and individuals with a history of abdominal surgery. In a resource-constrained healthcare environment, better patient outcomes and management may be achieved by directing resources towards higher-yield interventions.
